# Supplementary material for: Integrated multi-omics mapping of mitochondrial dysfunction and substrate preference in Barth syndrome cardiac tissue
Source: EMBO Mol Med. 2025 Oct 13;17(11):3227–46. doi: 10.1038/s44321-025-00320-5 (PMC12603102; doi:10.1038/s44321-025-00320-5)
Supplement: Supplementary file 9 — Expanded View Figures [file 44321_2025_320_MOESM9_ESM.pdf]

Expanded View Figures

Figure EV1. Proteomics overview of BTHS ($n = 5$; 2 collected at autopsy, 3 at transplantation) and young male non-failing donors ($n = 5$).

(**A**) Heatmap representing z-scores of the top 25 (ranked on *P*-value) proteins. (**B**) Volcano plot of mitochondrial proteins (according to MitoCarta 3.0), depicting depleted- (blue) and accumulated proteins (red) in cardiac tissue of BTHS individuals ($n = 5$) compared to young male non-failing donors ($n = 5$). Statistical comparisons were performed using moderated t-tests from the limma package in R, with Benjamini–Hochberg correction. Exact *p*-values corresponding to all statistical comparisons are provided in: Dataset EV4 (STATISTICS LIPIDOMICS), Dataset EV5 (STATISTICS METABOLOMICS), and Dataset EV6 (STATISTICS PROTEOMICS).

© The Author(s)

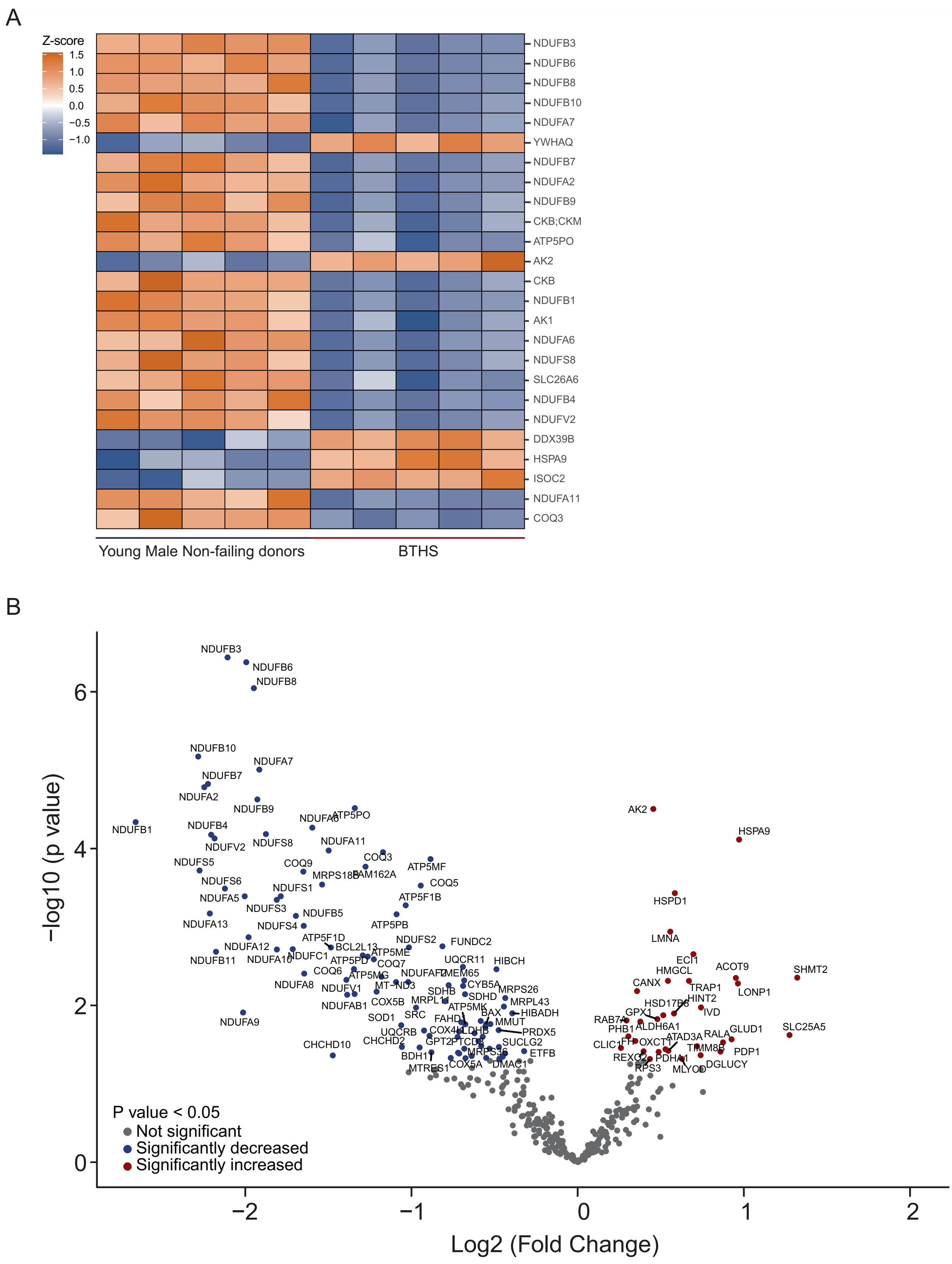
A
Z-score
1.5
1.0
0.5
0.0
−0.5
−1.0
NDUFB3
NDUFB6
NDUFB8
NDUFB10
NDUFA7
YWHAQ
NDUFB7
NDUFA2
NDUFB9
CKB;CKM
ATP5PO
AK2
CKB
NDUFB1
AK1
NDUFA6
NDUFS8
SLC26A6
NDUFB4
NDUFV2
DDX39B
HSPA9
ISOC2
NDUFA11
COQ3
Young Male Non-failing donors
BTHS
B
−log10 (p value)
Log2 (Fold Change)
0
2
4
6
−2
−1
0
1
2
P value < 0.05
Not significant
Significantly decreased
Significantly increased
NDUFB3
NDUFB6
NDUFB8
NDUFB10
NDUFA7
NDUFB7
NDUFA2
NDUFB9
NDUFB1
NDUFB4
NDUFV2
NDUFS8
NDUFA6
ATP5PO
NDUFA11
NDUFS5
COQ9
COQ3
MRPS18B
FAM162A
ATP5MF
NDUFS6
NDUFS1
COQ5
NDUFA5
NDUFS3
NDUFB5
ATP5F1B
NDUFA13
NDUFS4
ATP5PB
NDUFA12
ATP5F1D
BCL2L13
NDUFS2
FUNDC2
NDUFB11
NDUFA10
NDUFC1
ATP5ME
ATP5PD
COQ7
UQCR11
HIBCH
COQ6
ATP5MG
NDUFAF2
TMEM65
NDUFA8
NDUFV1
MT−ND3
CYB5A
SDHB
MRPS26
NDUFAB1
COX5B
MRPL14
SDHD
MRPL43
ATP5MK
NDUFA9
SOD1
SRC
FAHD1
BAX
HIBADH
MMUT
UQCRB
COX4I1
LDHB
PRDX5
CHCHD10
CHCHD2
GPT2
PTCD3
SUCLG2
BDH1
MRPS36
ETFB
MTRES1
COX5A
DMAC1
AK2
HSPA9
HSPD1
LMNA
ECI1
HMGCL
ACOT9
SHMT2
CANX
TRAP1
LONP1
HSD17B8
HINT2
GPX1
IVD
RAB7A
PHB1
ALDH6A1
GLUD1
SLC25A5
RALA
ATAD3A
CLIC1
FH
OXCT1
TIMM8B
PDP1
REXO2
PDHA1
RPS3
MLYCD
DGLUCY

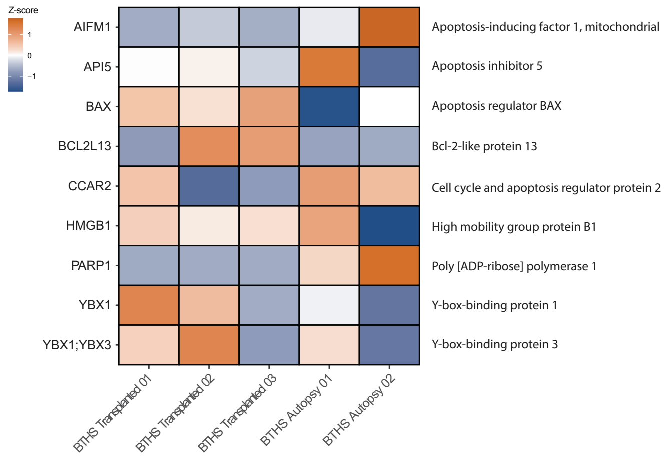

Figure EV2. Apoptosis/Necrosis in BTHS cardiac tissue.

Heatmap representing z-scores of an apoptosis/necrosis proteomics subset, alphabetically ordered. BTHS samples ($n = 5$; 2 collected at autopsy, 3 at transplantation) were compared between autopsy and transplantation.

© The Author(s)
